# Supplementary material for: Deletion of wfs1 Impairs Oligodendrocyte Precursor Cells Dorsal Distribution and Myelination Through the wfs1-hmgcs1 Axis in Zebrafish
Source: Biology (Basel). 2026 Aug 16;15(16):1402. doi: 10.3390/biology15161402 (PMC13510151; doi:10.3390/biology15161402)
Supplement: Supplementary file 1 [file biology-15-01402-s001.zip › File S1-Transcriptome sequencing data and differentially expressed genes (DEGs) in wfs1-- zebrafish at 5 dpf/2.KEGG/wfs1KOvsWT/UP/src/dre03320.html]

dre03320


- dre:334529

- Up regulated genes

334529(2.3128923028917)

- dre:406473

- Up regulated genes

406473(3.55746093515464)

- dre:553512

- Up regulated genes

553512(1.25576293759894)
- dre:550432

- Up regulated genes

550432(2.52614578061563)

- dre:553512

- Up regulated genes

553512(1.25576293759894)
- dre:550432

- Up regulated genes

550432(2.52614578061563)

- dre:140615

- Up regulated genes

140615(1.00910734824653)

- dre:30355

- Up regulated genes

30355\_1(2.05008708614496)

- dre:550134

- Up regulated genes

550134\_1(1.8446004641876)
- dre:777766

- Up regulated genes

novel.905(1.76535773054974)

Close
